# PIVKA‐II combined with tumor burden score to predict long‐term outcomes of AFP‐negative hepatocellular carcinoma patients after liver resection

**DOI:** 10.1002/cam4.6835

**Published:** 2023-12-21

**Authors:** Zhan‐cheng Qiu, You‐wei Wu, Wei‐li Qi, Chuan Li

**Affiliations:** ^1^ Division of Liver Surgery, Department of General Surgery, West China Hospital Sichuan University Chengdu Sichuan Province China

**Keywords:** AFP‐negative hepatocellular carcinoma patients, liver resection, prognostic scoring model, survival outcomes

## Abstract

**Background:**

This study aimed to establish a simple prognostic scoring model based on tumor burden score (TBS) and PIVKA‐II to predict long‐term outcomes of α‐fetoprotein (AFP)‐negative hepatocellular carcinoma (HCC) patients.

**Methods:**

511 patients were divided into the training cohort (*n* = 305) and the validation cohort (*n* = 206) at a ratio of 6:4. Receiver operating characteristic curves (ROC) were established to identify cutoff values of TBS and PIVKA‐II. Kaplan–Meier curves were used to analyze survival outcomes. The multivariable Cox regression was used to identify variables independently associated with survival outcomes. The predictive performance of the TBS‐PIVKA II score (TPS) model was compared with Barcelona clinic liver cancer (BCLC) stage and American Joint Committee on Cancer (AJCC TNM) stage.

**Results:**

The present study established the TPS model using a simple scoring system (0, 1 for low/high TBS [cutoff value: 4.1]; 0, 1 for low/high PIVKA‐II [cutoff value: 239 mAU/mL]). The TPS scoring model was divided into three levels according to the summation of TBS score and PIVKA‐II score: TPS 0, TPS 1, and TPS 2. The TPS scoring model was able to stratify OS (training: *p* < 0.001, validation: *p* < 0.001) and early recurrence (training: *p* < 0.001; validation: *p* = 0.001) in the training cohort and the validation cohort. The TPS score was independently associated with OS (TPS 1 vs. 0, HR: 2.28, 95% CI: 1.01–5.17; TPS 2 vs. 0, HR: 4.21, 95% CI: 2.01–8.84) and early recurrence (TPS 1 vs. 0, HR: 3.50, 95% CI: 1.71–7.16; TPS 2 vs. 0, HR: 3.79, 95% CI: 1.86–7.75) in the training cohort. The TPS scoring model outperformed BCLC stage and AJCC TNM stage in predicting OS and early recurrence in the training cohort and the validation cohort. But the TPS scoring model was unable to stratify the late recurrence in the training cohort (*p* = 0.872) and the validation cohort (*p* = 0.458).

**Conclusions:**

The TPS model outperformed the BCLC stage and AJCC TNM stage in predicting OS and early recurrence of AFP‐negative HCC patients after liver resection, which might better assist surgeons in screening AFP‐negative HCC patients who may benefit from liver resection.

## 1 INTRODUCTION

Hepatocellular carcinoma is a highly heterogeneous malignancy and is the fourth leading cause of tumor‐related death worldwide.^1^, ^2^ Surgical intervention is essential for HCC patients to attain long‐term survival. However, it is important to note that HCC patients who undergo liver resection have a very significant risk of disease recurrence (40%–70% in 5 years), meaning that a large proportion of patients will face retreatments after recurrence.^3^, ^4^ Thus, developing a preoperative risk stratification strategy to accurately screen HCC patients who will benefit the most from surgery is essential.

The prognosis of HCC patients after liver resection is associated with many factors, such as tumor burden (max tumor size and tumor number), pathological features (e.g., low differentiation, microvascular invasion, and capsular invasion), and serum tumor biomarkers (e.g., α‐fetoprotein and protein induced by vitamin K antagonist (PIVKA)‐II).^5^, ^6^, ^7^, ^8^ Some traditional HCC staging systems (e.g., Barcelona clinic liver cancer (BCLC) stage and American Joint Committee on Cancer (AJCC TNM) stage) based on tumor burden are currently being used to guide clinical practice.^9^, ^10^ However, some studies have shown that random binary cutoffs for both tumor size and number can reduce the statistical power.^11^, ^12^ Sasaki K et al. first proposed the tumor burden score (TBS) obtained by specific calculations after treating tumor size and number as continuous variables and demonstrated that TBS might be an accurate instrument for stratifying the prognosis of colorectal liver metastases patients undergoing resection.^13^ Further studies have also shown that TBS can forecast the prognosis of HCC patients undergoing hepatectomy.^7^, ^14^ However, TBS does not reflect the biological features of tumors because it contains only morphological characteristics. Serum α‐fetoprotein (AFP) is a typical tumor biomarker of HCC and plays a crucial function in monitoring, diagnosing, and determining the prognosis of HCC patients.^15^, ^16^, ^17^ Thus, whether combine AFP and TBS could predict survival outcomes of HCC patients after liver resection is an interesting topic, and several studies have demonstrated the synergistic effect of both in predicting the postoperative prognosis of HCC patients.^12^, ^18^, ^19^, ^20^

However, it is worth noting that AFP (at a threshold level of 20 ng/mL) has a limited ability to detect HCC (sensitivity of 40%–60% with specificity of 80–90%), which means that a proportion of HCC patients have negative AFP levels (< 20 ng/mL) at diagnosis, especially for small HCC patients.^21^ Protein induced by vitamin K deficiency II (PIVKA‐II) was identified for the first time in 1968 and was demonstrated to be a potential biomarker supplementing AFP for the diagnosis of HCC in many studies.^21^, ^22^, ^23^ Meanwhile, many studies also showed that preoperative serum PIVKA‐II levels were related to the prognosis of HCC patients after hepatectomy.^8^, ^24^, ^25^ Therefore, for preoperative AFP‐negative (<20 ng/mL) HCC patients, we hypothesized that the combination of PIVKA‐II and TBS had a strong prognostic effect. No study has investigated the synergistic impact of PIVKA‐II and TBS on long‐term outcomes of AFP‐negative HCC patients after liver resection so far. As a result, this present study examined the association between TBS‐PIVKA‐II score (TPS) and the prognosis (overall survival/early recurrence/late recurrence) of AFP‐negative HCC patients following liver resection.

## 2 MATERIALS AND METHODS

### 2.1 Patients

**FIGURE 1:**
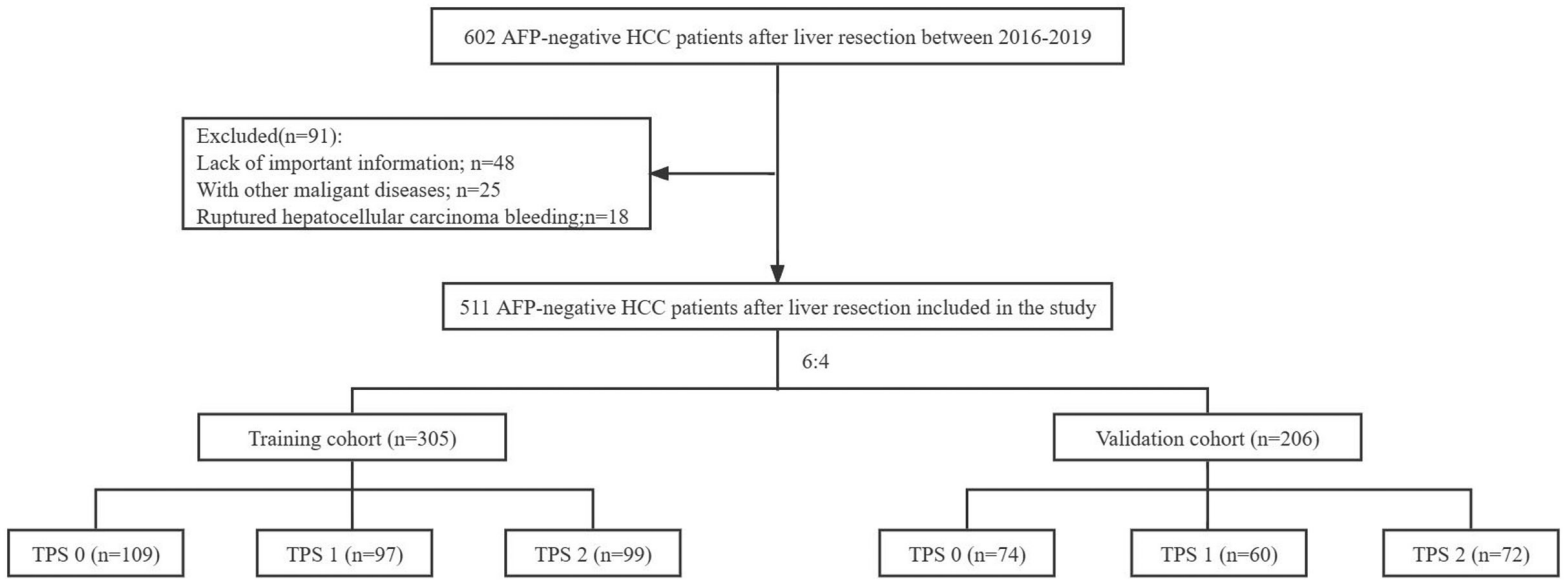
Flow chart for inclusion of patients in this study.

In our study, 602 AFP‐negative HCC patients with BCLC 0/A/B who received R0 resection at West China Hospital (WCH) between January 2016 and May 2019 were enrolled. Patients who met any of the following criteria were excluded from the study: (1) lack of important information; (2) with other malignant diseases; (3) ruptured hepatocellular carcinoma bleeding. Finally, 511 AFP‐negative HCC patients after R0 resection were included in our study for further analysis (Figure 1). Within 1 week prior to surgery, all laboratory tests were performed. All baseline information of patients in our study was retrospectively collected from WCH's Hospital Information System (HIS).

### 2.2 Outcomes

All patients were monitored 1 month after liver resection, every 3 months for the first 2 years, and then every 6 months. Follow‐up period‐based imaging protocols included abdominal ultrasound, enhanced CT, and MRI. Overall survival (OS) was the primary outcome of our study. OS was defined as the time from surgery to death from any cause or the last follow‐up (May 31, 2022). Early recurrence and late recurrence were the secondary outcomes of our study. Recurrence within 2 years of liver resection was defined as early recurrence. Early recurrence was defined as recurrence within 2 years following liver resection. Recurrence after 2 years after liver resection was considered late recurrence.^26^ Positive imaging results that were compared to the values from the preoperative exam or if they were verified by biopsy or resection were considered positive recurrence.^27^

### 2.3 Definitions

**FIGURE 2:**
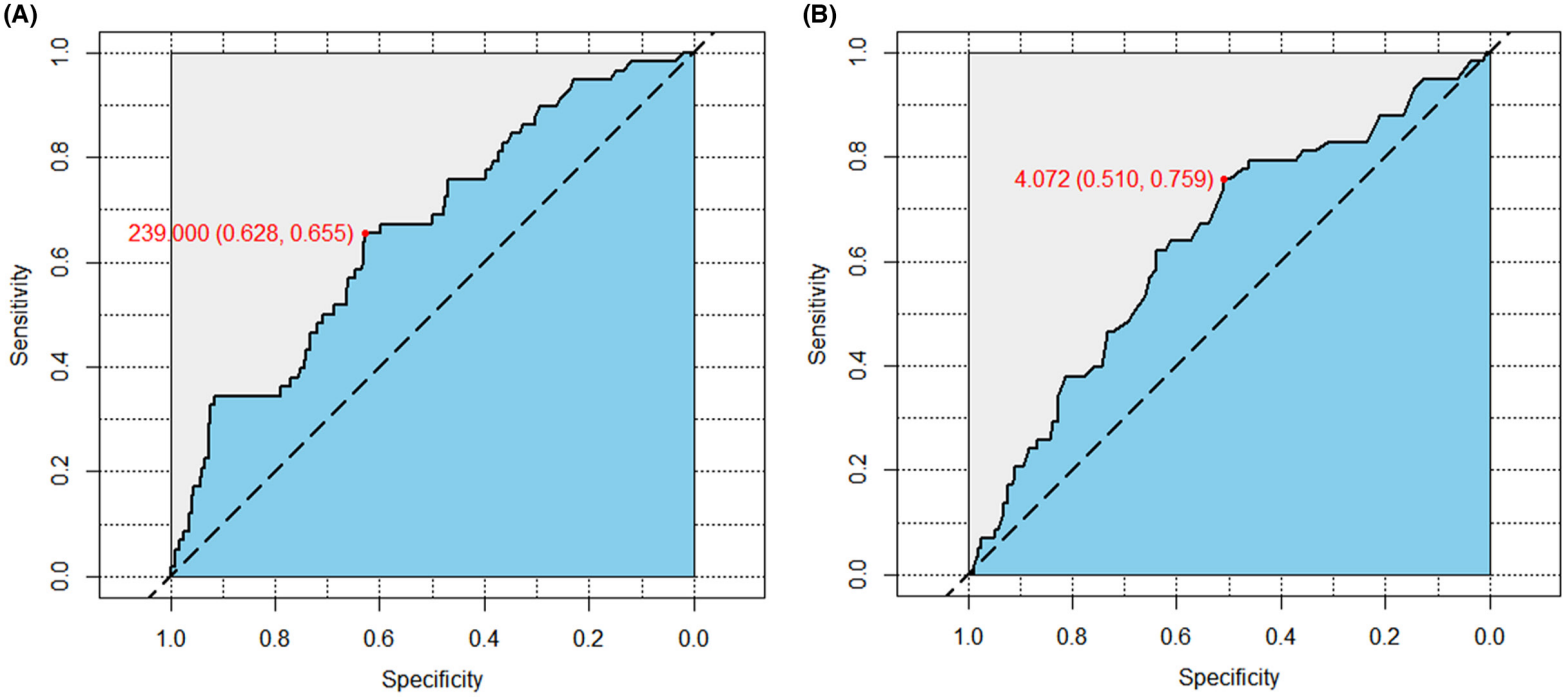
The cutoff values of TBS and PIVKA‐II determined by ROC curves. (A) PIVKA‐II; (B) TBS.

The CCI score was defined as the Charlson comorbidity index and was equal to the sum of the comorbidity scores.^28^ HBV‐related was defined as HCC caused by hepatitis B virus. ALBI grades were classified into three levels (Grades I, II, and III = ≤−2.60, <−2.60 to ≤−1.39, and > −1.39) based on the ALBI score (ALBI score = [(log10 bilirubin (in μmol/L) × 0.66) + (albumin (in g/L) × −0.085)]). Preoperative PIVKA‐II levels were separated into two grades based on the cutoff value calculated by receiver operating characteristic curves (ROC): (1) high level, >239.0 mAU/mL; (2) low level, ≤239.0 mAU/mL (Figure 2A). The tumor burden score (TBS) was calculated using the following equation: TBS^2^ = maximum tumor size^2^ + tumor number^2^.^29^ The ROC analysis was also used to determine the TBS cutoff value: (1) high level, >4.1; (2) low level, ≤4.1 (Figure 2B). The TBS level and the PIVKA‐II level were both assigned a score of 0–1 from low to high, respectively. TBS‐PIVKA II (TPS) score were the summation of TBS score and PIVKA‐II score, and the TPS score ranged from 0 to 2. Milan criteria was defined as up to three HCC nodules, the largest <3 cm in diameter or a single HCC nodule up to 5 cm in diameter.^30^ Major hepatectomy was defined as was defined as resection of more than three contiguous Couinaud segments.^31^ MVI was defined as microvascular invasion.

### 2.4 Statistical analyses

Patients were divided into the training cohort (*n* = 305) and the validation cohort (*n* = 206) at a ratio of 6:4 for the internal validation. Continuous variables were described as the means ± SDs or median and quartile. The analysis of variance or Kruskal–Wallis test was utilized to compare continuous variables between different TPS score groups in the training cohort and validation cohort. Categorical variables were described in numbers and percentages, and chi‐squared tests or Fisher's exact tests were used for comparison of categorical variables. Receiver operating characteristic (ROC) curves based on overall survival (OS) were established to identify the cutoff values of TBS and PIVKA‐II in the training cohort. The Kaplan–Meier method was used to compare the differences in OS, early recurrence, and late recurrence by the log‐rank test. Variables independently associated with OS and early recurrence were determined by univariable and multivariable Cox regression analyses using the stepwise method. The predictive ability of TPS was compared with that of BCLC stage, AJCC TNM stage by using Harrell's concordance index (C‐index), Akaike information criteria (AIC), and the area under the ROC curve (AUC). DeLong's test was used to compare the efficacy of ROC curves. Homogeneity was evaluated by the −2 log‐likelihood ratio (−2LLR) derived from the Cox regression model. Version 4.2.3 of the R statistical software was used for statistical analyses. *p* values <0.05 were statistically significant by the two‐tailed tests.

## 3 RESULTS

### 3.1 Patient characteristics

Patients were divided into the training cohort (*n* = 305) and the validation cohort (*n* = 206) at a ratio of 6:4 for internal validation (Figure 1). Variables were similar between the training cohort and the validation cohort (Table 1, all *p* > 0.05). Patients were separately divided into three groups in the training cohort (TPS 0, *n* = 109; TPS 1, *n* = 97; and TPS 2, *n* = 99) and the validation cohort (TPS 0, *n* = 74; TPS 1, *n* = 60; and TPS 2, *n* = 72) according to the TPS score. Table 2 presents the characteristics of patients in the three groups (Table 2). In the training cohort, patients in the TPS 0 group were younger (*p* = 0.033) and had more comorbidities (*p* = 0.010). Patients in the TPS 0 group all met with Milan criteria (*p* < 0.001) and were more likely to receive laparoscopic hepatectomy (*p* < 0.001), and patients in the TPS 2 group had larger tumor sizes (*p* < 0.001) and were more likely to receive major hepatectomy (*p* < 0.001). A larger proportion of patients in the TPS 2 group had capsular invasion (*p* = 0.001) and MVI (*p* < 0.001) in pathology, but more patients in the TPS 0 group experienced cirrhosis (*p* = 0.001). There were no significant differences among the training cohort's groups in the other variables (Table 2). In the validation cohort, patients in the TPS 0 group all met the Milan criteria (*p* < 0.001), and patients in the TPS 2 group had larger tumor sizes (*p* < 0.001) and were more likely to undergo major hepatectomy (*p* < 0.001). Meanwhile, patients in the TPS 2 group had worse pathological characteristics: a larger proportion of capsule invasion (*p* < 0.001) and MVI (*p* < 0.001).

**TABLE 1:** Clinicopathological characteristics of the entire cohort.

Variables	Training cohort (*n* = 305)	Validation cohort (*n* = 206)	*p* value
Age, years	57.4 ± 10.7	55.9 ± 11.0	0.131
Male	273 (89.5%)	182 (88.3%)	0.681
CCI score			0.705
1	180 (59.0%)	121 (58.7%)	
2	39 (12.8%)	22 (10.7%)	
≥3	86 (28.2%)	63 (30.6%)	
ALBI I/II	265/40	174/32	0.441
HBV‐related	286 (93.8%)	199 (96.6%)	0.153
PIVKA‐II, mAU/mL	158 (42,753)	148 (39,1233)	0.737
Low, ≤239	175 (57.4%)	116 (56.3%)	0.811
High, >239	130 (42.6%)	90 (43.7%)	
Max tumor size, cm	4.0 (3.0,5.7)	4.3 (2.8,6.0)	0.990
Single tumor	290 (95.1%)	193 (93.7%)	0.497
Tumor burden score	4.2 (3.2,5.9)	4.4 (3.0,6.1)	0.973
Low, ≤4.1	140 (45.9%)	92 (44.7%)	0.782
High, >4.1	165 (54.1%)	114 (55.3%)	
BCLC 0/A/B	25/269/11	22/174/10	0.475
AJCC TNM stage			0.643
I A/I B/II/III A	25/265/8/7	22/171/8/5	
Milan criteria	194 (63.6%)	126 (61.2%)	0.576
Laparoscopic/open hepatectomy	87/218	66/140	0.395
Major hepatectomy	77 (27.6%)	61 (29.6%)	0.276
Low differentiation	25 (8.2%)	13 (6.3%)	0.425
Capsular invasion	130 (42.6%)	97 (47.1%)	0.319
MVI	63 (20.7%)	45 (21.8%)	0.747
Cirrhosis	155 (50.8%)	108 (52.4%)	0.721

*Note*: ALBI grades were classified into three levels (Grades I, II, and III = ≤−2.60, <−2.60 to ≤−1.39, and > −1.39) based on the ALBI score (ALBI score = [(log10 bilirubin (in μmol/L) × 0.66) + (albumin (in g/L) × −0.085)]). Major hepatectomy was defined as was defined as the resection of more than three contiguous Couinaud segments. Milan criteria was defined as up to three HCC nodules, the largest <3 cm in diameter or a single HCC nodule up to 5 cm in diameter.

Abbreviations: AJCC TNM stage, American Joint Committee on Cancer; BCLC, Barcelona clinic liver cancer staging system; CCI, Charlson comorbidity index; HBV‐related, hepatitis B‐related hepatocellular carcinoma; MVI, microvascular invasion; PIVKA‐II, protein induced by vitamin K deficiency II.

**TABLE 2:** Characteristics of patients stratified by the TPS score.

	Training group (*n* = 305)				Validation group (*n* = 206)			
Variables	TPS 0 (*n* = 109)	TPS 1 (*n* = 97)	TPS 2 (*n* = 99)	*p* value	TPS 0 (*n* = 74)	TPS 1 (*n* = 60)	TPS 2 (*n* = 72)	*p* value
Age, years	55.7 ± 10.1	59.6 ± 11.4	57.1 ± 10.4	0.033	55.3 ± 9.5	55.4 ± 12.3	57.0 ± 11.2	0.251
Male	96 (88.1%)	86 (88.7%)	91 (91.9%)	0.629	63 (85.1%)	54 (90.0%)	65 (90.3%)	0.559
CCI score				0.010				0.523
1	60 (55.0%)	50 (51.5%)	70 (70.7%)		41 (55.4%)	35 (58.3%)	45 (62.5%)	
2	10 (9.2%)	18 (18.6%)	11 (11.1%)		6 (8.1%)	9 (15.0%)	7 (9.7%)	
≥3	39 (35.8%)	29 (29.9%)	18 (18.2%)		27 (36.5%)	16 (26.7%)	20 (27.8%)	
ALBI I/II	92/17	89/8	84/15	0.227	66/8	53/7	55/17	0.063
HBV‐related	106 (97.2%)	91 (93.8%)	89 (89.9%)	0.091	71 (95.9%)	58 (96.7%)	70 (97.2%)	0.913
Max tumor size, cm	2.9 (2.3,3.3)	4.2 (3.5,5.7)	6.1 (5.0,8.6)	<0.001	2.5 (2,3.2)	4.7 (3.6,5.6)	6.2 (5.0,8.6)	<0.001
Single tumor	106 (97.2%)	93 (95.9%)	91 (91.9%)	0.188	71 (95.9%)	55 (91.7%)	67 (93.1%)	0.557
Milan criteria	109 (100%)	60 (61.9%)	25 (25.3%)	<0.001	74 (100%)	35 (58.3%)	17 (23.6%)	<0.001
Laparoscopic/Open hepatectomy	45/64	25/72	17/82	<0.001	30/44	17/43	19/53	0.143
Major hepatectomy	10 (9.2%)	25 (25.8%)	42 (42.4%)	<0.001	11 (14.9%)	12 (12.0%)	38 (52.8%)	<0.001
Low differentiation	8 (7.3%)	8 (8.2%)	9 (9.1%)	0.899	6 (8.1%)	1 (1.7%)	6 (8.3%)	0.144
Capsular invasion	41 (37.6%)	32 (33.0%)	57 (57.6%)	0.001	21 (28.4%)	29 (48.3%)	47 (65.3%)	<0.001
MVI	12 (11.0%)	17 (17.5%)	34 (34.3%)	<0.001	8 (10.8%)	8 (13.3%)	29 (40.3%)	<0.001
Cirrhosis	68 (62.4%)	50 (51.5%)	37 (37.4%)	0.001	44 (59.5%)	30 (50.0%)	34 (47.2%)	0.303

Abbreviations: TPS, tumor burden score (TBS)‐PIVKA II score model.

### 3.2 Impact of TPS score on OS, early recurrence and late recurrence of AFP‐negative HCC patients after liver resection

**FIGURE 3:**
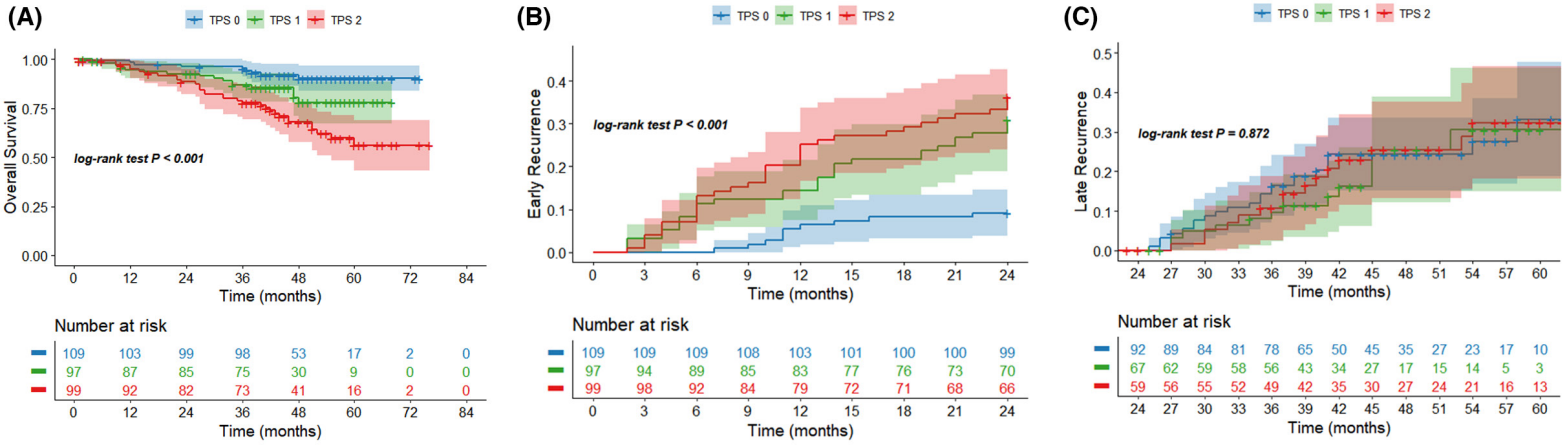
Kaplan–Meier curves for the survival outcomes in the training cohort. (A) Overall survival (OS); (B) early recurrence; (C) late recurrence.

In the training cohort, the median follow‐up time was 48.0 months, and the 5‐year overall survival rate was 74.0%. Patients were divided into three groups based on TPS score in the training cohort. Patients in the TPS 0 group had a 5‐year survival rate of 90.2%, patients in the TPS 1 group had a 5‐year survival rate of 77.9%, and patients in the TPS 2 group had a 5‐year survival rate of 56.0%. In the Kaplan–Meier analysis in our study, the TPS score had good performance in stratifying the OS of AFP‐negative HCC patients after liver resection (*p* < 0.001, Figure 3A). Meanwhile, during the follow‐up period, 127 patients developed recurrence, of which 76 patients developed early recurrence and 51 patients developed late recurrence in the entire training cohort. The cumulative incidence of early recurrence was significantly different among the three groups and patients in the TPS 0 group had the lowest cumulative incidence of early recurrence (*p* < 0.001, Figure 3B). However, the cumulative incidence of late recurrence was comparable among the three groups (*p* = 0.872, Figure 3C).

**FIGURE 4:**
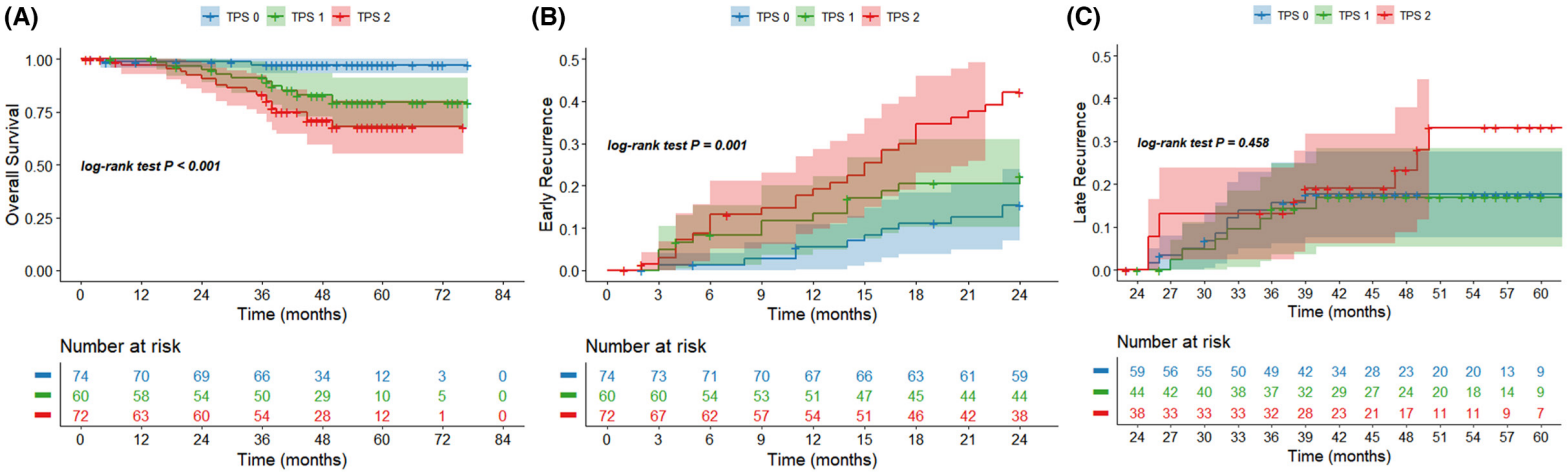
Kaplan–Meier curves for the survival outcomes in the validation cohort. (A) Overall survival (OS); (B) early recurrence; (C) late recurrence.

In the validation cohort, the median follow‐up time was 48 months, and the 5‐year overall survival rate was 81.6%. Patients were also divided into three groups based on TPS score in the validation cohort. Patients in the TPS 0 group had a 5‐year survival rate of 97.2%, patients in the TPS 1 group had a 5‐year survival rate of 79.5%, and patients in the TPS 2 group had a 5‐year survival rate of 67.8%. Significant differences were also observed in OS by Kaplan–Meier analysis (*p* < 0.001, Figure 4A). During the follow‐up period, 79 patients experienced recurrence in the validation cohort, of which 52 patients developed early recurrence and 27 patients developed late recurrence. There were still significant differences in the cumulative incidence of early recurrence (*p* = 0.001, Figure 4B) but no difference in the cumulative incidence of late recurrence (*p* = 0.458, Figure 4C).

### 3.3 Variables independently associated with OS and early recurrence

In our study, a multivariable Cox regression model was established to identify variables independently associated with OS and early recurrence in the training cohort. In the univariate analysis for OS, TPS score, TBS level, PIVKA‐II level, BCLC stage, AJCC TNM stage, tumor size, tumor number, Milan criteria, and MVI were potential predictors for OS (all *p* < 0.05, Table 3). These potential variables were included in the multivariable analysis. In our multivariable Cox regression model, patients in the TPS 1 group and the TPS 2 group both had a significantly higher risk of death than those in the TPS 0 group (TPS 1, HR: 2.28, 95% CI: 1.01–5.17, *p* = 0.048; TPS 2, HR: 4.21, 95% CI: 2.01–8.84, *p* < 0.001, Table 3). Multiple tumor (HR: 2.58, 95% CI: 1.10–6.07, *p* = 0.029, Table 3) was another independent variable associated with OS.

**TABLE 3:** Cox proportional hazards regression for factors associated with overall survival in the training cohort.

	Univariate analysis			Multivariate analysis		
Variables	HR	(95% CI)	*p* value	HR	(95% CI)	*p* value
TPS						
0	Ref					
1	2.30	1.02–5.21	0.046	2.28	1.01–5.17	0.048
2	4.45	2.13–9.30	<0.001	4.21	2.01–8.84	<0.001
TBS level						
Low, ≤4.1	Ref					
High, >4.1	2.96	1.63–5.41	<0.001	‐	‐	‐
PIVKA II level						
Low, ≤239 mAU/mL	Ref					
High, >239 mAU/mL	2.65	1.54–4.55	<0.001	‐	‐	‐
BCLC stage	2.72	1.16–6.39	0.021	‐	‐	‐
AJCC TNM stage	1.80	1.09–2.99	0.023	‐	‐	‐
Max tumor size, >5 cm	1.96	1.17–3.27	0.011	‐	‐	‐
Single tumor						
Yes	Ref					
No	3.27	1.40–7.64	0.019	2.58	1.10–6.07	0.029
Milan criteria						
Yes	Ref					
No	2.32	1.38–3.90	0.001	‐	‐	‐
MVI	2.04	1.16–3.60	0.020	‐	‐	‐

Table 4 shows the variables associated with early recurrence in the training cohort. In the univariate analysis for early recurrence, TPS score, TBS level, PIVKA‐II level, BCLC stage, AJCC TNM stage, tumor size, tumor number, Milan criteria, and MVI were potential predictors for early recurrence. In our multivariable Cox regression model, patients in the TPS 1 group and the TPS 2 group were both more likely to experience early recurrence than those in the TPS 0 group (TPS 1, HR: 3.50, 95% CI: 1.71–7.16, *p* = 0.001; TPS 2, HR: 3.79, 95% CI: 1.86–7.75, *p* < 0.001, Table 4). Other independent variables associated with early recurrence included multiple tumor (HR: 2.65, 95% CI: 1.20–5.86, *p* = 0.016) and MVI (HR: 2.32, 95% CI: 1.42–3.79, *p* = 0.001, Table 4).

**TABLE 4:** Cox proportional hazards regression for factors associated with early recurrence in the training cohort.

	Univariate analysis			Multivariate analysis		
Variables	HR	(95% CI)	*p* value	HR	(95% CI)	*p* value
TPS						
0	Ref					
1	3.71	1.81–7.58	<0.001	3.50	1.71–7.16	0.001
2	4.73	2.35–9.54	<0.001	3.79	1.86–7.75	<0.001
TBS level						
Low, ≤4.1	Ref					
High, >4.1	2.95	1.76–4.97	<0.001	‐	‐	‐
PIVKA II level						
Low, ≤239 mAU/mL	Ref					
High, >239 mAU/ml	2.15	1.36–3.40	0.001	‐	‐	‐
BCLC stage	3.67	1.86–7.18	<0.001	‐	‐	‐
AJCC TNM stage	1.96	1.31–2.93	0.001	‐	‐	‐
Max tumor size, >5 cm	2.33	1.47–3.67	<0.001	‐	‐	‐
Single tumor						
Yes	Ref					
No	2.53	1.16–5.51	0.019	2.65	1.20–5.86	0.016
Milan criteria						
Yes	Ref					
No	2.63	1.67–4.14	<0.001	‐	‐	‐
MVI	2.60	1.63–4.17	<0.001	2.32	1.42–3.79	0.001

### 3.4 Predictive performance of the TPS model

**FIGURE 5:**
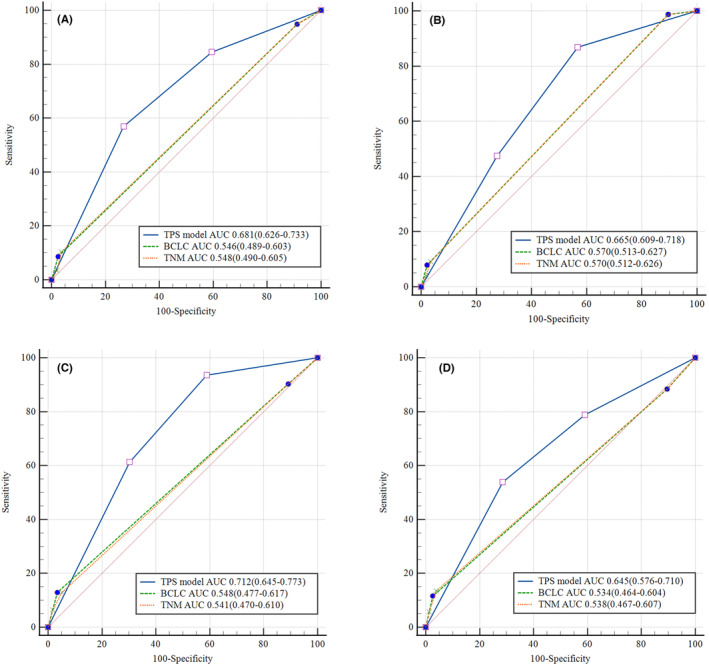
Receiver operating characteristic curve analysis comparing prognostic discrimination between the TPS model and other staging systems in the training and the validation cohort. (A) The ability of predicting OS in the training cohort; (B) the ability of predicting early recurrence in the validation cohort; (C) the ability of predicting OS in the validation cohort; (D) the ability of predicting early recurrence in the validation cohort.

In our study, the ability of the TBS‐PIVKA II score model to predict OS and early recurrence was compared with that of the BCLC stage and AJCC TNM stage in the training cohort and the validation cohort. In the training cohort, the AUCs for OS were 0.681 (95% CI: 0.626–0.733), 0.546 (95% CI: 0.489–0.603), and 0.548 (95% CI: 0.490–0.605) for the TPS model, BCLC stage, and AJCC TNM stage, respectively (Figure 5A). Significant differences were observed in the TPS model versus BCLC stage (*p* < 0.001) and the TPS model versus AJCC TNM stage (*p* < 0.001). The AUCs for early recurrence were 0.665 (95% CI: 0.609–0.718), 0.570 (95% CI: 0.513–0.627), and 0.570 (95% CI: 0.512–0.626) for the TPS model, BCLC stage, and AJCC TNM stage, respectively (Figure 5B). The TPS model also outperformed BCLC stage (*p* = 0.003) and AJCC TNM stage (*p* = 0.005) in predicting early recurrence. In terms of both early recurrence and OS, the TPS model (OS, AIC: 571.9; early recurrence, AIC: 694.4) had smaller AIC values than BCLC stage (OS, AIC: 585.1; early recurrence, AIC: 706.5) and AJCC TNM stage (OS, AIC: 587.4; early recurrence, AIC: 710.1, Table 5). Meanwhile, the TPS model had a higher C‐index (OS, C‐index: 0.647, 95% CI: 0.580–0.713; early recurrence, C‐index: 0.634, 95% CI: 0.565–0.702) than BCLC stage (OS, C‐index: 0.543, 95% CI: 0.495–0.592; early recurrence, C‐index: 0.566, 95% CI: 0.532–0.601) and AJCC TNM stage (OS, C‐index: 0.547, 95% CI: 0.495–0.599; early recurrence, C‐index: 0.566, 95% CI: 0.530–0.602, Table 5). And the TPS model showed higher homogeneity (OS, −2LLR: 603.4; early recurrence, −2LLR: 821.1) than BCLC stage (OS, −2LLR: 617.9; early recurrence, −2LLR: 829.5) and AJCC TNM stage (OS, −2LLR: 618.8; early recurrence, −2LLR: 833.8, Table 5).

**TABLE 5:** Comparison of prognostic performances of the TPS model, BCLC stage, and AJCC TNM stage.

Models	C‐Index	95% CI	Akaike information criterion (AIC)	Homogeneity (−2LLR)
Overall survival				
Training cohort				
TPS model	0.647	0.580–0.713	571.9	603.4
BCLC stage	0.543	0.495–0.592	585.1	617.9
AJCC TNM stage	0.547	0.495–0.599	587.4	618.8
Validation cohort				
TPS model	0.697	0.618–0.775	282.7	297.1
BCLC stage	0.549	0.471–0.626	296.3	311.5
AJCC TNM stage	0.542	0.464–0.620	300.4	311.9
Early recurrence				
Training cohort				
TPS model	0.649	0.595–0.703	694.4	821.1
BCLC stage	0.566	0.532–0.601	706.5	829.5
AJCC TNM stage	0.566	0.530–0.602	710.1	833.8
Validation cohort				
TPS model	0.634	0.565–0.702	461.5	521.9
BCLC stage	0.535	0.474–0.597	466.7	532.2
AJCC TNM stage	0.538	0.474–0.603	469.5	531.1

Abbreviation: ‐2LLR, ‐2 log‐likelihood ratio.

We further validated the performance of the TPS model in predicting OS and early recurrence in the validation cohort. In the validation cohort, the AUC for the TPS model in predicting OS was 0.712 (95% CI: 0.645–0.773), which was significantly better than that for BCLC stage (AUC: 0.548, 95% CI: 0.477–0.617, *p =* 0.001) and AJCC TNM stage (AUC: 0.541, 95% CI: 0.470–0.610, *p* < 0.001, Figure 5C). The AUC for the TPS model in predicting early recurrence was 0.645 (95% CI: 0.576–0.710), which was also significantly better than that for BCLC stage (AUC: 0.534, 95% CI: 0.464–0.604, *p* = 0.013) and AJCC TNM stage (AUC: 0.538, 95% CI: 0.467–0.607, *p* = 0.023, Figure 5D). Compared with BCLC stage and AJCC TNM stage, the TPS model also had a lower AIC value and a higher C‐index in predicting OS and early recurrence (Table 5). Moreover, the TPS model also showed higher homogeneity in predicting OS and early recurrence in the validation cohort (Table 5). Thus, the TPS model also outperformed BCLC stage and AJCC TNM stage in the validation cohort (Table 5).

### 3.5 Performance of the TPS model in the subgroup of patients with or without cirrhosis

In the subgroup of AFP‐negative HCC patients with cirrhosis after liver resection, the TPS model was also able to stratify OS and early recurrence (OS: *p* < 0.001; early recurrence: *p* = 0.007; Figure S1). The AUCs for OS were 0.697 (95% CI: 0.638–0.752), 0.546 (95% CI: 0.484–0.607), and 0.546 (95% CI: 0.483–0.607) for the TPS model, BCLC stage, and AJCC TNM stage, respectively (Figure S2A). Significant differences were observed in the TPS model versus BCLC stage (*p* < 0.001) and the TPS model versus AJCC TNM stage (*p* < 0.001). The AUCs for early recurrence were 0.616 (95% CI: 0.554–0.675), 0.569 (95% CI: 0.507–0.629), and 0.575 (95% CI: 0.512–0.635) for the TPS model, BCLC stage, and AJCC TNM stage, respectively (Figure S2B), but results of the DeLong's test showed that there seemed no statistic difference in the ability of the TPS model in predicting early recurrence in the AFP‐negative HCC patients with cirrhosis after liver resection compared with BCLC stage (*p* = 0.266) and AJCC TNM stage (*p =* 0.318). In predicting early recurrence, although the TPS model had a higher C‐index, it did not exhibit dominance in terms of AIC and homogeneity (Table S1).

In the subgroup of AFP‐negative HCC patients without cirrhosis after liver resection, the TPS model continued to demonstrate its efficacy in effectively stratifying OS (*p* < 0.001) and early recurrence (*p* < 0.001, Figure S3). The AUCs for OS were 0.707 (95% CI: 0.646–0.763), 0.555 (95% CI: 0.491–0.618), and 0.550 (95% CI: 0.486–0.613) for the TPS model, BCLC stage, and AJCC TNM stage, respectively (Figure S4A). Significant differences were observed in the TPS model versus BCLC stage (*p* < 0.001) and the TPS model versus AJCC TNM stage (*p* < 0.001). The AUCs for early recurrence were 0.712 (95% CI: 0.651–0.767), 0.542 (95% CI: 0.478–0.605), and 0.537 (95% CI: 0.473–0.600) for the TPS model, BCLC stage, and AJCC TNM stage, respectively (Figure S2B). The TPS model also outperformed BCLC stage (*p* < 0.001) and AJCC TNM stage (*p* < 0.001) in predicting early recurrence (Figure S4B). The TPS model was still preferable compared with BCLC stage and AJCC TNM stage in predicting OS and early recurrence due to its higher C‐index, lower AIC, and higher homogeneity (Table S1).

## 4 DISCUSSION

Liver resection is an essential therapy option for HCC patients in order to attain long‐term survival, but due to the high heterogeneity of HCC, the survival outcomes of HCC patients after liver resection may vary significantly.^14^ Thus, it is essential to establish a preoperative risk stratification strategy to select the best surgical candidate in HCC patients. Some traditional HCC staging systems (e.g., BCLC and AJCC TNM stage) that currently are used for guiding the clinical treatment strategy are focused on the tumor morphological behaviors of HCC and ignore the biological behaviors of HCC, which results in some controversies in screening HCC patients suitable for surgery.^32^ Therefore, some studies investigated the synergetic impact of morphological characteristics and biological characteristics (tumor markers, e.g., AFP) on long‐term outcomes of HCC patients after hepatectomy and demonstrated the good performance of the combination of TBS and AFP in stratifying the OS of HCC patients after liver resection.^12^, ^18^, ^19^, ^33^, ^34^ However, there is no relevant study in AFP‐negative HCC patients after liver resection. The present study investigated the relationship between the synergetic impact of combining TBS and PIVKA‐II and survival outcomes of AFP‐negative HCC patients within BCLC 0/A/B after liver resection. We established a simple prognostic scoring model (TPS model) based on TBS and preoperative PIVKA‐II levels and demonstrated that the TPS model had excellent performance in stratifying OS and early recurrence in AFP‐negative HCC patients after liver resection. Meanwhile, the TPS score remained an independent risk factor for OS and early recurrence after adjusting for confounding factors. Our study also demonstrated that the TPS model outperformed the BCLC stage and AJCC TNM stage in predicting OS and early recurrence of AFP‐negative HCC patients after liver resection, which might better assist surgeons in screening AFP‐negative HCC patients who may benefit from liver resection. However, the ability of the TPS model to stratify late recurrence of AFP‐negative HCC patients needs to be further studied.

Traditional staging systems (e.g., BCLC stage and AJCC TNM stage) that consider tumor size and number as dichotomous variables are currently used to guide clinical practice. However, employing arbitrary category cutoff values when analyzing continuous (tumor size) or ordinal (tumor number) data might reduce statistical power and result in incorrect causal inferences, for example, treatment modalities for HCC patients in BCLC stage B remain somewhat controversial.^35^, ^36^ Therefore, Sasaki K et al. first proposed tumor burden score (TBS, treat tumor size and number as continuous variables) to differentiate the prognosis of colorectal liver metastasis patients after surgery.^13^ The predictive value of TBS was then further confirmed in many solid tumors as well, including HCC.^37^, ^38^, ^39^ Tsilimigras DI et al. based on a multicenter retrospective study and demonstrated that TBS could stratify the OS of HCC patients within BCLC 0/A/B and may identify patients in BCLC B who maybe candidates for resection.^14^ Some studies also reported that TBS was independently associated with recurrence of HCC patients after liver resection.^7^, ^40^

In addition to tumor burden, which only contains morphological features, tumor biomarkers, which respond to the biological behavior of tumors, also play an important role in the prognosis of HCC patients after liver resection. AFP has been a well‐established tumor biomarker of HCC for decades. Many studies have shown that AFP is important not only for the early detection and diagnosis of HCC but also for the prognosis of HCC patients after hepatectomy.^41^, ^42^ However, current guidelines focus on tumor load and somewhat ignore the role of tumor biomarkers in prognosis. Thus, some interesting studies investigated the relationship of combining TBS and AFP with the prognosis of HCC patients after hepatectomy and demonstrated the excellent synergistic influence of TBS and AFP on survival outcomes of HCC patients after liver resection. Ding HF et al. developed a prediction model (ATS model) based on TBS and AFP to predict recurrence after HCC and demonstrated that its predictive ability was better than that of traditional staging systems (e.g., BCLC stage and AJCC TNM stage).^19^ For HCC patients after liver transplantation, Duvoux C et al. found that the AFP model was more accurate than the Milan criteria at predicting postoperative recurrence and mortality.^43^, ^44^ Thus, considering both tumor morphology and biological behavior may be an effective method for predicting HCC survival outcomes.

However, for AFP‐negative HCC patients, how can we combine morphology and biological behavior to stratify their survival outcomes after liver resection? PIVKA‐II has been demonstrated to be a potential biomarker supplementing AFP for the diagnosis of HCC and is associated with the prognosis of HCC patients after hepatectomy in many studies. Wang MD et al. based on a multicenter retrospective study reported that preoperative PIVKA‐II positivity, rather than AFP positivity, was independently related to early recurrence after HCC resection.^8^ Ota M et al. demonstrated preoperative the PIVKA‐II level was independently associated with the OS of HCC patients after hepatectomy.^45^ Therefore, based on relevant data in our medical center, our study first investigated the relationship between the combination of TBS and PIVKA‐II and survival outcomes of AFP‐negative HCC patients after liver resection. Although many studies have demonstrated the predictive value of TBS and PIVKA‐II in predicting prognosis of HCC patients, it is worth noting that there is no agreement on the cutoff values of TBS and PIVKA‐II in predicting prognosis of HCC patients because the cutoff values are strongly associated with the estimation cohort. As far as we know, there is no study to investigate the cutoff values of TBS and PIVKA‐II in the cohort of AFP‐negative HCC patients. Thus, we identified the cutoff values of TBS and PIVKA‐II based on ROC analysis and then developed a simple prognostic scoring model (TPS model) to stratify OS and early recurrence of AFP‐negative HCC patients after liver resection. The TPS model both had better performance in predicting OS and early recurrence compared with the BCLC staging system and AJCC TNM staging system. The superiority and simplicity of our model may make it a useful tool for identifying surgical candidates among AFP‐negative patients. However, it is worth noting that the TPS model lacks the ability to stratify late recurrence of AFP‐negative HCC patients after liver resection. Late recurrence after surgery is usually regarded as the new development of tumors of multicentric origin, mainly associated with underlying liver diseases.^26^ From this perspective, the inability of the TPS model to stratify for late recurrence seems to be expected. Compared with late recurrence, early recurrence after surgery mainly is associated with the aggressiveness of the primary tumor. Some factors representing tumor aggressiveness such as tumor size, tumor number, MVI, and low differentiation have been shown to correlate with early recurrence in many studies.^26^ Preoperative biological markers (e.g., AFP and PIVKA‐II) were also demonstrated to be associated with early recurrence of HCC patients after hepatectomy.^8^, ^46^ In the present study, patients in the higher TBS‐PIVKA II score group had larger tumor sizes and were more likely to experience MVI. Poté N et al. also demonstrated that the high PIVKA‐II level was independently associated with MVI.^24^ These findings, to some extent explained the result that the TPS model could stratify the early recurrence of AFP‐negative HCC patients after liver resection.

In our subgroup analysis, the TPS model still showed superior performance in predicting the OS of AFP‐negative HCC patients with or without cirrhosis after liver resection and also showed superior performance in predicting early recurrence of AFP‐negative HCC patients without cirrhosis after liver resection. However, the TPS model did not seem to show superiority in predicting early recurrence in the subgroup of AFP‐negative HCC patients with cirrhosis after liver resection. In the subgroup analysis of AFP‐negative HCC patients with cirrhosis after liver resection, the TPS model had a higher AUC and a higher C‐index in predicting early recurrence, but the results of the DeLong's test showed no statistical significance. Thus, the ability of predicting early recurrence in the AFP‐negative HCC patients after liver resection with cirrhosis needs to be further studied although the TPS model is not inferior to traditional stage systems (BCLC and AJCC TNM stage). HCC patients with cirrhosis usually have varying degrees of hepatic dysfunction. Good liver function reserve is necessary for liver resection, and the Albumin‐Bilirubin score helps to further stratify stage A of the Child‐Pugh score and has been demonstrated to be associated with early recurrence of HCC patients after liver resection.^6^ This to some extent explain why our model did not show superiority in predicting early recurrence of AFP‐negative HCC patients with cirrhosis after liver resection. Thus, in the future study about predicting early recurrence of AFP‐negative HCC patients with cirrhosis after liver resection, evaluation of liver function may be essential.

Some excellent predictive models have been developed to predict the prognosis of HCC patients, such as the up‐to‐seven criteria, the ERASL‐pre model. The up‐to‐seven criteria was initially proposed as an extension of Milan criteria for selecting HCC patients for liver transplantation and its role in guiding the treatment of HCC patients with intermediate or advanced stage has been further demonstrated.^47^, ^48^, ^49^ Recently Vitale A et al. reported that the up‐to‐seven criteria seemed to have similar predictive value compared with TBS based on the analysis of a large cohort of HCC patients received different treatments.^11^ The up‐to‐seven criteria was mainly used to help making treatment decisions, such as TACE, systemic therapy, for intermediate or advanced HCC patients in many studies.^48^, ^49^ Thus, it seemed difficult for us to investigate the relationship between our TPS model and the up‐to‐seven criteria, whether it was because of the retrospective nature of our study or because our study mainly included early‐stage HCC patients, but it may be an interesting topic for our future research. Early recurrence after liver resection in HCC patients is a constant concern for surgeons. Chan AWH et al. established a model called “ERASL‐pre model,” which consisted of male, age, ALBI grade, AFP, tumor size, and tumor number, to predict early recurrence after liver resection in HCC patients and the model showed a good performance.^6^ This study demonstrated evaluation of preoperative liver function might be important for assessing early recurrence of HCC patients after liver resection. In our subgroup analysis of AFP‐negative HCC patients with cirrhosis after liver resection, although our model also could stratify early recurrence, it did not show enough superiority compared with BCLC stage and AJCC TNM stage. Maybe the inclusion of liver function in further studies may improve the predictive power of our model.

There were several limitations in our study. First, our study was a single‐center retrospective study. Thus, there may be some selection bias in our study. Multicenter studies and even prospective investigations are required to further confirm our findings. Second, although our study included HCC patients of BCLC B, the number of patients with BCLC stage B was low. Moreover, the updated version (2022) of the Barcelona Clinic Liver Cancer (BCLC) classification system categorizes patients in the BCLC‐B stage into three distinct groups based on the assessment of tumor load and liver function.^9^ Thus, the performance of our model in predicting prognosis of AFP‐negative patients with BCLC‐B stage needs to be further demonstrated in the future studies based on the updated BCLC stage system. Third, our study only included AFP‐negative HCC patients of BCLC 0, BCLC A and BCLC B. For HCC patients with advanced tumor (BCLC C) or AFP‐positive, our results also need to be further demonstrated by further studies.

## 5 CONCLUSIONS

Our study demonstrated the important role of the combination of TBS and PIVKA‐II in predicting long‐term outcomes of AFP‐negative HCC patients. This simple TBS‐PIVKA II score model developed in our study had excellent performance in predicting OS and early recurrence of AFP‐negative HCC patients within BCLC 0/A/B after liver resection, which might better assist surgeons in screening AFP‐negative HCC patients who may benefit from liver resection.

## AUTHOR CONTRIBUTIONS

**Zhan‐cheng Qiu:** Conceptualization (equal); data curation (equal); formal analysis (lead); methodology (equal); resources (equal); software (lead); writing – original draft (lead). **You‐wei Wu:** Data curation (equal); formal analysis (equal); methodology (equal); resources (equal); writing – original draft (equal). **Wei‐li Qi:** Data curation (equal); formal analysis (equal); methodology (equal). **Chuan Li:** Conceptualization (equal); formal analysis (equal); methodology (equal); supervision (lead); writing – original draft (equal); writing – review and editing (lead).

## FUNDING INFORMATION

This study did not receive any funding or support from the private sector. The authors were solely responsible for data interpretation and presentation.

## CONFLICT OF INTEREST STATEMENT

No financial conflict or other relationships for each author to be declared.

## ETHICAL APPROVAL STATEMENT

The West China Hospital's Ethics Committee gave its approval to this study. The committee decided not to require informed consent because the study was retrospective.

## Supporting information


Figure S1.

Figure S2.

Figure S3.

Figure S4.

Table S1.

## Data Availability

The corresponding author will, upon reasonable request, share the datasets used and/or analyzed in the present research.
