# Supplementary figures and images for: PIVKA‐II combined with tumor burden score to predict long‐term outcomes of AFP‐negative hepatocellular carcinoma patients after liver resection
Source: Cancer Med. 2023 Dec 21;13(1):e6835. doi: 10.1002/cam4.6835 (PMC10807584; doi:10.1002/cam4.6835)

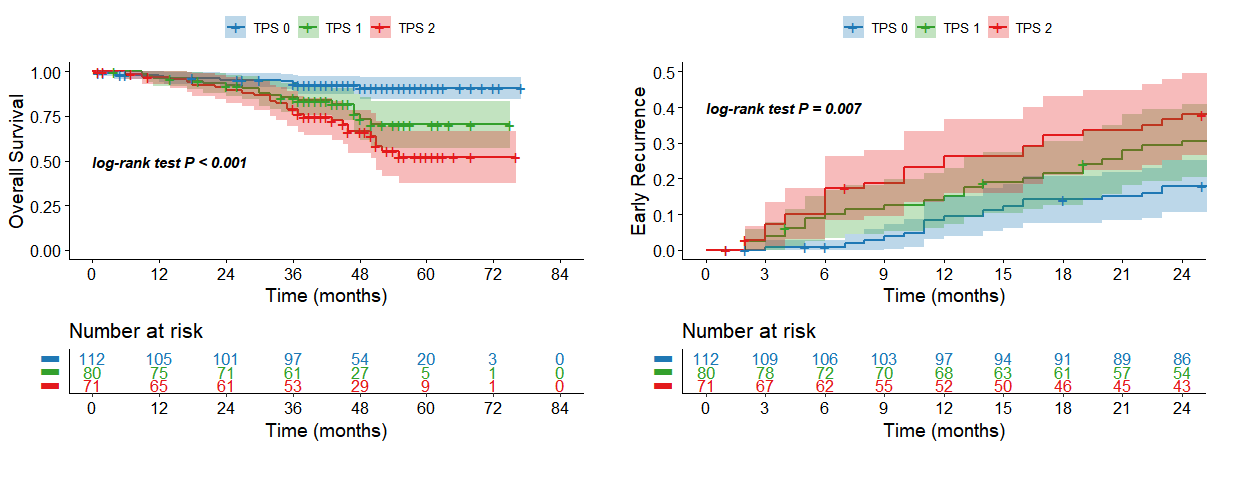

Supplement: Supplementary file 1 — Figure S1. [file CAM4-13-e6835-s001.tif]

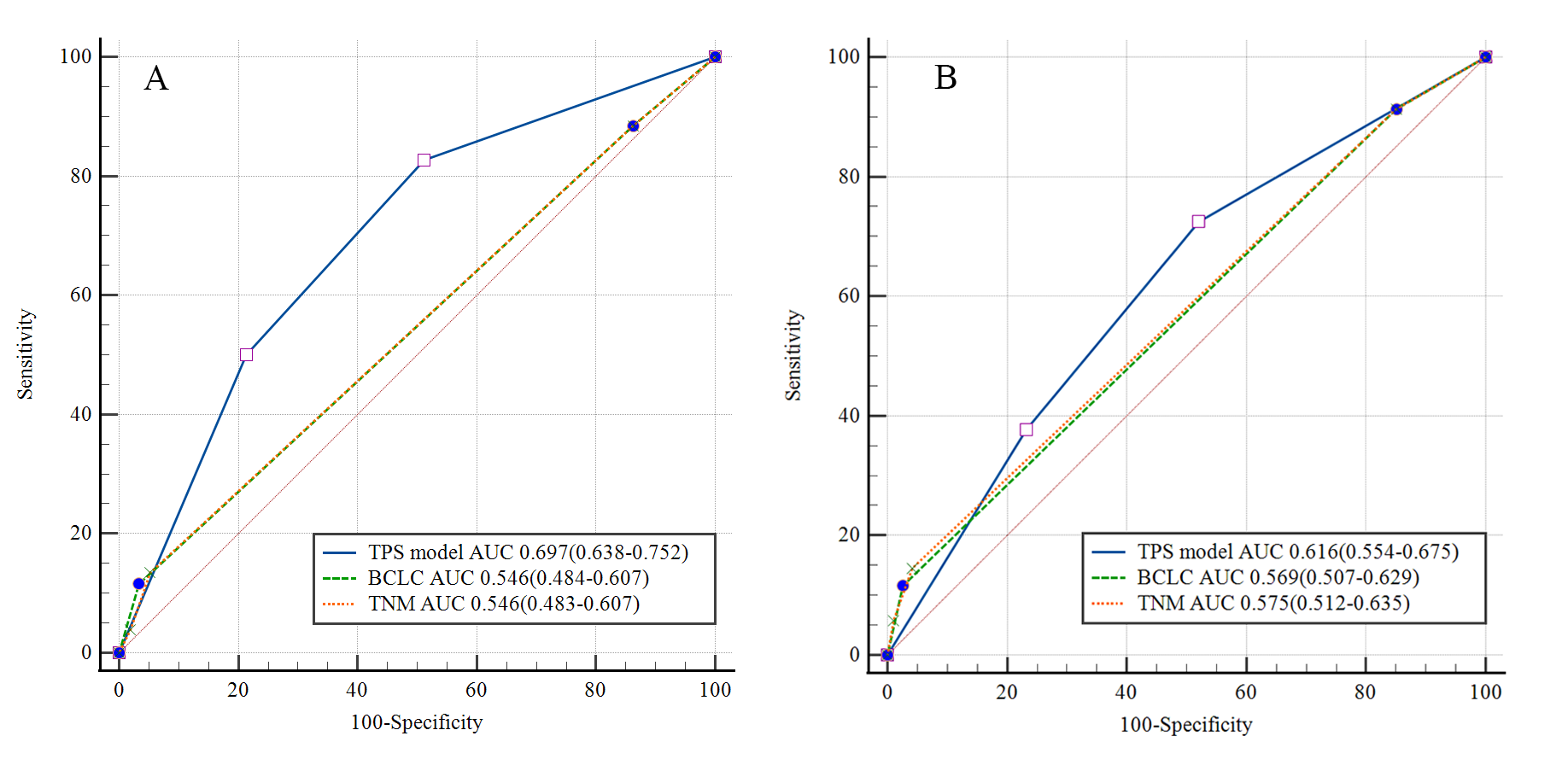

Supplement: Supplementary file 2 — Figure S2. [file CAM4-13-e6835-s005.tif]

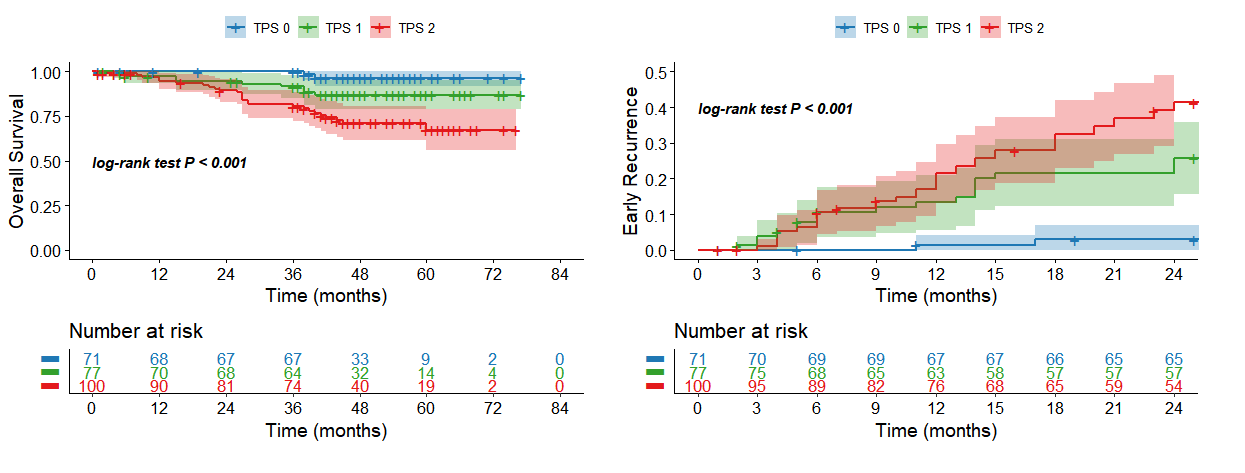

Supplement: Supplementary file 3 — Figure S3. [file CAM4-13-e6835-s004.tif]

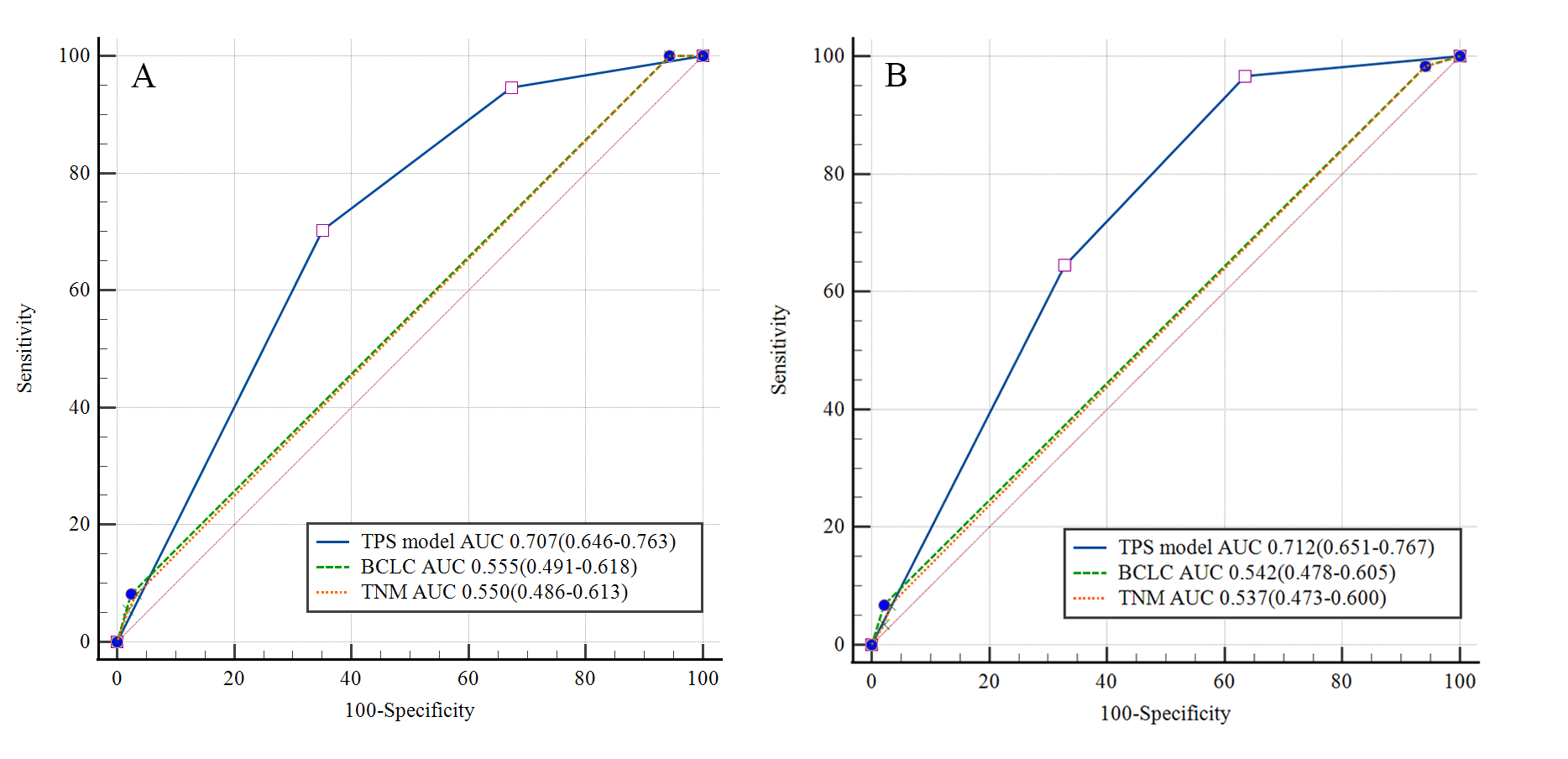

Supplement: Supplementary file 4 — Figure S4. [file CAM4-13-e6835-s003.tif]
