# Supplementary material for: PIVKA‐II combined with tumor burden score to predict long‐term outcomes of AFP‐negative hepatocellular carcinoma patients after liver resection
Source: Cancer Med. 2023 Dec 21;13(1):e6835. doi: 10.1002/cam4.6835 (PMC10807584; doi:10.1002/cam4.6835)
Supplement: Supplementary file 5 — Table S1. [file CAM4-13-e6835-s002.docx]

| Models | C-Index | 95% CI | Akaike information criterion (AIC) | Homogeneity (-2LLR) |
| --- | --- | --- | --- | --- |
| **Non-cirrhosis group** |  |  |  |  |
| **Overall survival** |  |  |  |  |
| TPS model | 0.692 | 0.627-0.757 | 370.8 | 369.1 |
| BCLC stage | 0.553 | 0.510-0.596 | 384.8 | 383.1 |
| AJCC TNM stage | 0.549 | 0.506-0.592 | 387.2 | 385.5 |
| **Early recurrence** |  |  |  |  |
| TPS model | 0.688 | 0.633-0.743 | 598.5 | 597.7 |
| BCLC stage | 0.539 | 0.500-0.578 | 624.6 | 623.6 |
| AJCC TNM stage | 0.534 | 0.495-0.573 | 627.5 | 626.4 |
| **Cirrhosis group** |  |  |  |  |
| **Overall survival** |  |  |  |  |
| TPS model | 0.651 | 0.578-0.724 | 523.3 | 521.7 |
| BCLC stage | 0.541 | 0.476-0.606 | 540.3 | 538.5 |
| AJCC TNM stage | 0.544 | 0.477-0.611 | 540.4 | 538.7 |
| **Early recurrence** |  |  |  |  |
| TPS model | 0.609 | 0.546-0.672 | 735.6 | 734.6 |
| BCLC stage | 0.568 | 0.519-0.617 | 736.5 | 735.5 |
| AJCC TNM stage | 0.572 | 0.527-0.623 | 735.0 | 734.0 |

**Supplementary Table S1:** Prognostic performances of the TPS model in the subgroup of cirrhosis (non- cirrhosis and cirrhosis).
